# Evaluating Swine Injection Technologies as a Workplace Musculoskeletal Injury Intervention: A Study Protocol

**DOI:** 10.1155/2017/5094509

**Published:** 2017-10-29

**Authors:** Catherine Trask, Brenna Bath, Stephan Milosavljevic, Aaron M. Kociolek, Bernardo Predicala, Erika Penz, Olugbenga Adebayo, Lee Whittington

**Affiliations:** ^1^Canadian Centre for Health and Safety in Agriculture, College of Medicine, University of Saskatchewan, 104 Clinic Place, P.O. Box 23, Saskatoon, SK, Canada S7N 2Z4; ^2^School of Physical Therapy, College of Medicine, University of Saskatchewan, Suite 3400, 104 Clinic Place, Saskatoon, SK, Canada S7N 2Z4; ^3^School of Physical and Health Education, Nipissing University, 100 College Drive, Box 5002, North Bay, ON, Canada P1B 8L7; ^4^Prairie Swine Centre Inc., 2105-8th St. East, P.O. Box 21057, Saskatoon, SK, Canada S7H 5N9; ^5^Department of Medicine, College of Medicine, University of Saskatchewan, 103 Hospital Drive, Saskatoon, SK, Canada S7N 0W8

## Abstract

Intensification of modern swine production has led to many new technologies, including needleless injectors. Although needleless injectors may increase productivity (by reducing injection time) and reduce needlestick injuries, the effect on risk for musculoskeletal disorders is not clear. This project will compare conventional needles with needleless injectors in terms of cost, productivity, injury rates, biomechanical exposures, and worker preference. Muscle activity (EMG) and hand/wrist posture will be measured on swine workers performing injection tasks with both injection methods. Video recordings during the exposure assessments will compare the duration and productivity for each injection method using time-and-motion methods. Injury claim data from up to 60 pig barns will be analyzed for needlestick and musculoskeletal injuries before/after needleless injector adoption. Workers and managers will be asked about what they like and dislike about each method and what helps and hinders successful implementation. The information above will be input into a cost-benefit model to determine the incremental effects of needleless injectors in terms of occupational health, worker preference, and the financial “bottom line” of the farm. Findings will be relevant to the swine industry and are intended to be transferable to other new technologies in animal production.

## 1. Background

A recent review of musculoskeletal disorders (MSD) among farmers reported 91% lifetime prevalence for any type of MSD and one-year prevalence of 77% [1]. MSD in agriculture impact worker productivity and quality of life. Sprains and strains account for 28% of the approximately 200,000 time loss injuries on US farms, and 43% of all agricultural injuries are categorized as “overexertions” or MSD [2]. Livestock agriculture introduces unique risks; a study of Swedish pig and dairy farms found the 12-month prevalence of any MSD to be 78% in swine workers, with the most common injuries being in the upper extremities (62%) and the back (57%) [3]. Danish swine workers were also found to have high rates of MSD [4]. A recent pilot study of workers in the Canadian swine industry found a 12-month prevalence of 92% of MSD and 58% of respondents reported having their activities interrupted by MSD symptoms [5]. Such disorders are the most common cause of work absence in self-employed Dutch farmers [6], and Irish farm income is shown to be lower when operators have MSD-related disability [7].

Agricultural MSD occur within a changing industrial context. Between 2001 and 2011, the number of pig farms in Canada decreased from 15,472 to 7,371 (~52% decrease), demonstrating the overall trend of consolidating pork production into larger barns [8, 9]. This trend is seen in other industrialized nations such as Sweden [3]. Combined with globalized commodity markets' downturn in pork prices [10], this creates a context of very low profit margins and high production pressures. It has been noted in other industries that when the primary motivations are driven by economics, global competition, and production, Occupational Health and Safety (OH&S) and ergonomics may be seen as a threat or regulatory barrier rather than as a benefit [11]. It is therefore imperative to consider business needs when pursuing OH&S goals.

Economic pressures also drive technological advancement, especially in those areas that will enhance productivity or reduce production costs. For example, needleless injectors are now available to replace hypodermic needle injectors. They may help reduce needlestick injuries in workers, although the possibility of accidental injection into workers remains, as does the possibility of abscess at the injection site [12]. Needleless injector devices can eliminate broken needle contamination in meat and may also increase productivity. However, the repetitive, forceful gripping required during needleless injecting, in addition to other postural or repetitive strain due to task specialization, may introduce new hazards and greater risk of musculoskeletal injury. There is evidence that industrial intensification and its process changes may either increase existing MSD exposures or introduce new ones. In dairy farming, industrial intensification has been shown to change MSD exposure profiles [8, 10], as well as increase [13] or change the location [14] of reported MSD (e.g., from the knees to the back). Swedish dairy workers, for example, demonstrated increasing rates of MSD from 83% in 1988 to 90% in 2002 [13]. This change was concurrent with increased task time and musculoskeletal exposure duration using modern milking equipment [15]. As livestock intensification may carry increased risk, further research is needed to ensure that technological advances take worker health and safety into account while assessing and acknowledging economic factors.

The need for ergonomic interventions that limit musculoskeletal risk factors in agriculture (and particularly animal handling work) is widely acknowledged [16, 17]. There is evidence that ergonomic interventions can be cost-effective for a business, but the quality of economic evaluations is frequently poor and usually does not describe the economic benefits [18]. Despite agreement that a higher quality of ergonomic intervention research is required [19], most intervention studies focus solely on musculoskeletal exposure with and without modified tools or equipment during lab-based or simulated work [20–23], or in small field studies [24, 25].

Injection for nutritional supplements, immunizing animals, and providing treatment to sick animals is a standard practice in the swine industry (and livestock production in general). Historically, barn workers performed injections using a hypodermic needle and injecting (subcutaneous or intramuscular) into the animals. However, an alternative needleless method uses pressure to force a jet of vaccine or other liquid through the dermal layer and into the subcutaneous tissue [26]. Although needleless injection was developed for humans nearly a century ago, its use is not widespread due to patient preference [27]. The technology has also been slow to transfer to agriculture. A recent review of needleless injection methods notes that it eliminates broken needles in meat (enhancing food safety of subsequent pork products), needlestick injury in workers, and needle disposal; delivers a more consistent, lower dose of vaccine; and causes less stress in animals [26]. There is also a suggestion of greater immunological effects using needleless injectors [26], although this finding is inconsistent [28]. However, the review authors also note several disadvantages of needleless systems: substantial equipment purchasing costs; exhaust gas infrastructure for pneumatic devices; increased training and maintenance needs; and worker preference for a known method (i.e., needle injection) [26].

Although needleless injectors eliminate needlestick injuries and needle contamination of meat (benefits), they may introduce an unintended consequence of increased forearm activity, frequency of gripping motion, and awkward postures (costs in terms of injury claims and lost productivity). Repetitive movements during work tasks have been consistently shown to increase risk of musculoskeletal disorders [29, 30]. Such movements may be intensified on industrialized farms. A study of modern, intensive pork production observed hand grip frequencies of 30 per minute during “piglet processing” (an injection-intensive task) and between 10 and 15 per minute during “herd health checks” (which involves injections) [5]. This kind of repetitive and forceful gripping is a risk factor, specifically for upper limb musculoskeletal disorders including carpal tunnel syndrome [31]. Increases in gripping force and frequency with needleless injectors may negate the needlestick benefits, but an empirical comparison is required to make any conclusions. Needleless injectors' use is on the rise, but they are not implemented in all barns due to uncertainty about cost-benefit trade-offs. This makes needleless injectors an ideal current technology to evaluate. To our knowledge, no evaluation of needleless injectors has included worker health issues such as needlestick and musculoskeletal injuries as well as economic cost/benefits, a worrisome gap at a time when needleless injection is growing in popularity. To address these gaps, this project aims to investigate the implementation of needleless injectors in terms of cost, productivity, injury rates, biomechanical exposures, and worker preference.

## 2. Methods

**Figure 1:**
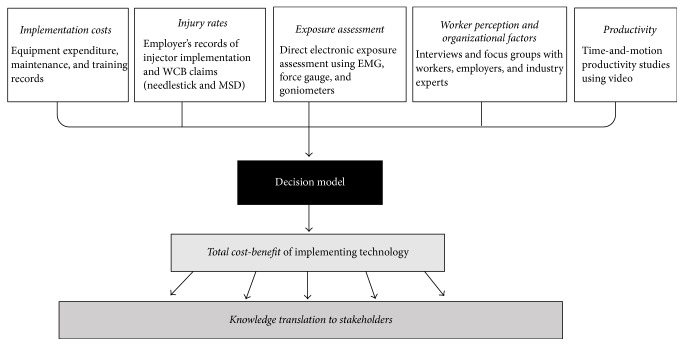
Research project diagram for developing a comprehensive decision-making toolkit for implementing new technologies in the Pork Value Chain. A related web tool will allow users to enter inputs for their situation and estimate the total cost-benefit.

This project will use a combination of quantitative and qualitative data collection strategies: (1) electronic measurement of muscle activity and hand/wrist postures; (2) time-and-motion productivity analysis of each method; (3) employer records of needlestick, upper limb, and other musculoskeletal injuries (i.e., claims data and incident reports); (4) worker focus groups and key informant interviews to determine barriers and facilitators to safe injection advantages of each method; and (5) an economic analysis of the comparative costs and benefits. A subsequent decision-making toolkit will be based on project outcomes as shown in Figure 1.

This project has been approved by the University of Saskatchewan research ethics board (certificate number Beh 16-161). All participation will be completely voluntary after seeking informed consent.

### 2.1. Costs of Implementation

The typical range of costs for each method will be assessed by querying research partners, with confirmation by producer records where needed. Relevant costs include equipment purchase, maintenance, consumable supplies, and worker training. Although we have secured participation of 60 barns in the Canadian provinces of Saskatchewan and 40 barns in Manitoba, we anticipate that costs will be fairly consistent due to the relatively low number of needleless injector systems available for purchase in North American, so only a 10% sample (6 barns) will be needed. If costs are very diverse, an additional 6 barns will be contacted.

### 2.2. Injury Records and Incident Reports

Injury records (i.e., WCB claims and incident reports, including claim costs) will be obtained from large, multisite pork producers, representing a population of 420 swine workers in Saskatchewan and 300 workers in Manitoba. These data are already collected in the course of business operations, with the addition of noting which barns have needleless injectors and the date they were introduced. All barn and personal identifiers will be anonymized before delivery to the research team.

### 2.3. Barn Measurement: Biomechanical Exposure and Productivity Assessment

**Figure 2:**
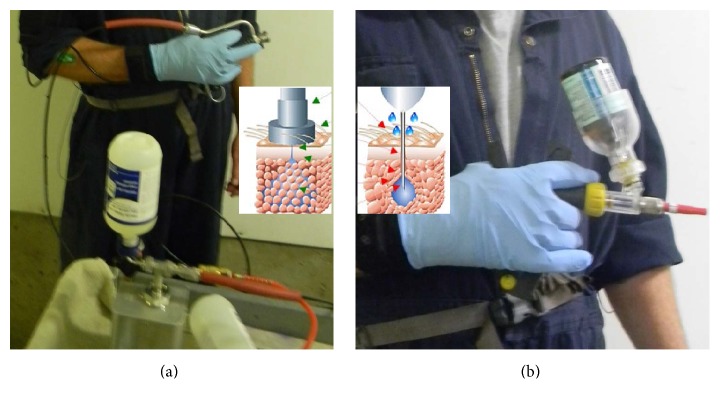
Swine injection methods for comparison in this study: (a) needleless injector powered by pneumatic compressor; (b) conventional needle injector.

Volunteer swine workers will be recruited from the swine production barn of the Prairie Swine Centre, a research-intensive barn with ties to the University of Saskatchewan. This study will measure both needleless injectors and conventional needle injectors (shown in Figure 2) for common swine tasks: piglet processing and weaning pig injection. Measurements will be conducted for at least 2 workers for each task and repeated for up to 100 performances of each task/injection method performance, yielding ~400 measurements in total. This design entails no need to match participants for age and sex; the repeated measurements with both injections methods will serve as their own controls. Although biosecurity limits the ability to use research equipment at multiple barns, it is anticipated that collecting measurements from a single barn will permit useful conclusions. Collecting these data for several hours over a variety of workers for 2 days each during different tasks allows for a robust estimate of the exposures and cycle times and also gives insight into how widely these times vary under different conditions.

Participant characteristics including sociodemographic variables, employment history, recent musculoskeletal pain or discomfort, and history of MSD will be collected by a questionnaire. Video of the workers using each injection method will be recorded for subsequent analysis using time-and-motion description to determine worker productivity. Wrist flexion-extension and radioulnar deviation angles and finger joint flexion angles of the dominant upper limb will be measured using an instrumented data glove (CyberGlove III, CyberGlove Systems, San Jose, CA). The data glove is made of stretchable fabric to accommodate a range of hand sizes, and the fingertips are open to enable routine tool use. The embedded sensors are thin and flexible to allow for unimpeded performance of work tasks. The data glove was previously used to assess kinematics during precision gripping [32] and hand tool usage [33, 34]. The glove will be connected to a receiver worn on a belt strap, which will wirelessly transmit all data to a laptop at 50 Hz. Wrist and finger joint velocities and acceleration will also be derived from the joint angles to quantify dynamics [35].

**Figure 3:**
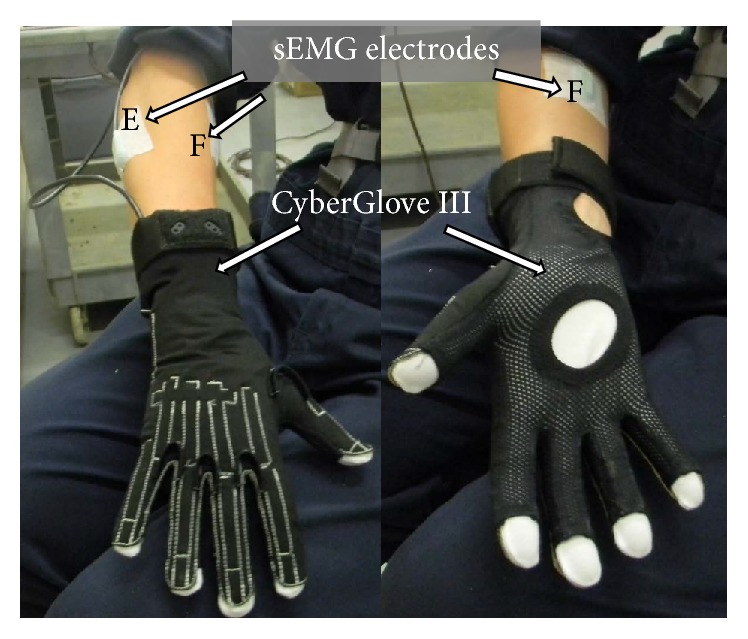
Biomechanical exposure data collection set-up with sEMG electrodes over extensor (E) and flexor (F) muscle groups and CyberGlove III.

To infer musculoskeletal loading, forearm flexor and extensor muscle activity will be collected via surface electromyography (sEMG). Bipolar surface electrodes with a fixed interelectrode distance of 20 mm (SX-230-1000, Biometrics Ltd., Newport, UK) will be positioned over the bulk of the forearm flexor compartment and the forearm extensor compartment (Figure 3). These electrode placements are consistent with previous research that demonstrated that a “through forearm setting” was optimal for assessing forearm loading while minimizing motion artefact due to forearm pronation and supination during hand-intensive tasks [36, 37]. Forearm flexor and extensor sEMG will be collected with a portable data-logger (MWX8, Biometrics, Ltd.) and digitally stored to a microSD card at 1000 Hz. Additionally, a digital trigger (IS3LED, Biometrics, Ltd.) will initiate each data recording, thus providing time-synchronized measurement of hand/wrist posture and forearm muscle activity simultaneously. The trigger will also be used to time-stamp each individual injection trial, providing a means to perform a matched cycle-to-cycle comparison between the needle and needleless injection methods. These methods will be pilot-tested and refined to ensure that they are practical and feasible with the selected tasks and in the barn environment.

### 2.4. Worker Perception and Organizational Factors: Management Interviews/Worker Focus Groups

Reduction of exposures does not guarantee a reduction in MSD [19]. Although many ergonomic intervention studies have shown decreases in exposure during short-term simulated work [38], long-term effectiveness may be limited by worker acceptance, equipment availability, or time constraints [39]. To address this, focus groups will be conducted with groups of 4–8 workers at barns that have made the switch from needle to needleless injectors. To capture some diversity in organizational culture, 10 barns will be invited to participate. Semistructured discussions will be led by a trained facilitator and will solicit feedback on the duration, difficulty, advantages, and disadvantages of each method. Employer perspectives from these barns will also be sought via manager interviews. Managers responsible for human resources, health and safety, and purchasing will be interviewed at the worksite regarding the nature of device use, maintenance, and training.

## 3. Data Analysis

### 3.1. Injury Records Analysis

Data are available both for in-barn incident reports and provincial Workers' Compensation Board claims (i.e., injury location, type, total time loss, claim costs, etc.) for the past 6 years; both of these sources will be investigated for both needlestick/accidental injection injuries and upper limb musculoskeletal injuries. Comparisons will be made before and after needleless injector implementation, with implementation for at least 15 months. The postimplementation injury rates will be calculated following a 3-month “implementation period” after needleless injectors are delivered to the barn. Injury data will be divided into equal-duration periods before and after the injectors were introduced. For example, where a barn that implemented needleless injectors 30 months ago, a 27-month period after intervention (30 months less the 3-month implementation period) would be compared to a 27-month period before intervention. The maximum duration for comparison will be 60 months after implementation and an unlimited preimplementation period. We anticipate approximately 40 barns being eligible, representing approximately 300 full-time equivalents (FTE) per year over the past 10 years (typical barn age) for a sample of 3000 FTE-years. Incident rates will be calculated for the pre- and postimplementation periods, and survival analysis will be performed to determine the highest-risk milieu for both needlestick and upper limb MSD. We hypothesize that needleless barns will have reduced needlestick injuries but increased upper limb claims.

### 3.2. Productivity Analysis

Time-and-motion analysis involves dividing a work task such as “injecting pigs” into subtasks, activities, and discrete movements or elements, using a stopwatch to time these elements and determine the total task time, cycle time (e.g., the time to inject a single pig), and the duty cycle (the proportion of time spent doing productive work) [40]. In addition to productivity analysis, any needlestick injuries that occur during video recording will be noted. Injection time per pig will be calculated and compared via repeated measures ANOVA with the factors “task type” (piglet processing and inoculations) and “injection type” (conventional needle or needleless).

### 3.3. Barn Exposure Analysis

EMG will be normalized (i.e., calibrated) to muscle-specific preshift maximum voluntary efforts and summarized via amplitude probability distribution function (APDF; 10th 50th, and 90th percentiles). Wrist and finger joint kinematics, including joint angles, angular velocities, and angular acceleration will also be summarized by APDF. In addition to joint kinematics and EMG, video-based task observation will be used to assess exposures and injury risk with standardized ergonomic tools for the upper limb: Revised Strain Index [41] and Hand Activity Level (HAL) [42]. These tools have the additional advantage of being inexpensive, widely accepted, and accessible to OH&S professionals in industry. Advanced biomechanical analysis methods will provide further means of comparison, including feeding wrist and finger joint kinematics and EMG into recently developed models to estimate carpal tunnel pressure [43, 44] as well as tendon travel [45, 46] and friction [47]. Analyses of all exposure metrics, including joint kinematics, muscle activity, pressures, tendon travel, and friction, will be analyzed using repeated measures ANOVA with the factors “task type” (piglet processing and inoculations) and “injection type” (conventional needle or needleless).

### 3.4. Management Interviews/Worker Focus Group Analysis

Focus groups and interviews will be audio recorded and transcribed and then given to participants to check for accuracy. This qualitative data will identify barriers and enablers of injector implementation success and identify any unintended consequences. In addition to developing a potential “wish list” of specifications for improved designs, this information will be used to identify pork industry specific organizational factors and “readiness for implementation” indicators. Worker focus group feedback and manager perceptions of advantages and disadvantages of each process will be analyzed qualitatively using an inductive thematic analysis approach [48] with NVivo qualitative analysis software.

### 3.5. Cost and Economic Assessment

A cost-benefit analysis of the needleless injector compared with current practice will be performed from the perspective of the swine producer over a 5-year time frame (based on estimated working life of needleless injector system). The costs of the needleless injection technique will be estimated and compared to standard practice of conventional needles in terms of capital costs (equipment), variable costs of production, absenteeism, and injury claims. The benefits of the needleless injector in terms of worker productivity will be estimated and expressed in monetary terms. The sum of the net present value of incremental costs and benefits of the needleless injector will be compared with current practice. The technology with the highest net present value will be considered the most cost-effective from the perspective of the swine producer. The costs and benefits will be used to inform a business case for employers considering implementing needleless injectors, including return on investment and estimates of enterprise scale to identify which producers (if any) will see a net benefit from the investment. This analysis will be performed using the pork production enterprise model developed by the Prairie Swine Centre [49–51]. The enterprise model is designed to evaluate the effective return that near-market research provides the Canadian pork industry. Results generated from specific research projects are entered and evaluated within the enterprise model with the final calculation being a change in return per pig marketed associated with the research project. By assessing various projects within a standard operation, Prairie Swine Centre is able to accurately assess the risk/reward trade-off to producers through the incorporation of new research outcomes and technologies.

*Developing the Decision Toolkit.* One of the major industry-relevant outcomes of this project will be the development of a decision-making toolkit capable of balancing the impact of multiple inputs: productivity, injury, and so forth. This tool will be translated into a web-based application where users can enter characteristics of their organization, current injury rates, anticipated implementation costs, and then receive an estimate of the cost-benefit that a certain innovation will deliver. The algorithm to estimate cost-benefit will be developed using the data collected during this project, as well as prioritizations and input from the stakeholder advisory group. To address situations where the nature of the proposed innovation differs substantially from the needleless injector case study, margins of error will also be estimated.

## 4. Knowledge Translation

To ensure that the results of this study remain relevant to the pork industry, the research team will maintain strong industry connections through the use the Canadian Institutes of Health Research integrated knowledge translation (KT) approach, engaging stakeholders throughout the research process [52]. Integrated KT is marked by the collaboration of researchers and knowledge users to shape the research process starting with research questions and methodology through to interpreting and disseminating results. Forming partnerships with key stakeholders to conduct this research produces results that are more relevant and more likely to be put into practice. This project involves ongoing collaborative interaction between decision-makers and researchers that will result in mutual learning through the process of planning, producing, disseminating, and applying existing or new research in decision-making. In keeping with this integrated KT philosophy, this proposal has been developed in collaboration with the Prairie Swine Centre, industry partners, the Saskatchewan Pork Development Board, and the Manitoba Pork Board. In addition to specific input during grant development, we will invite representatives to form a stakeholder advisory group to help inform and guide key stages in the research process.

## 5. Discussion

Intensification of Canadian pig farms [9] has resulted in labour context changes from small independent farms (where family members perform a variety of tasks) to an employer model (where workers perform a narrow range of tasks within specialized roles). This shift may further contribute to already high rates of musculoskeletal disorders among pig farmers [3, 4]. This intensification has potential to impact worker health in Manitoba, where 47% of all WCB claims are currently caused by overexertion or repetitive motion [53]. It also tips economic conditions to favour investment in specialized equipment, technology, and facilities. The context of intensified livestock operations may carry new risk factors for injury, but it also presents an opportunity to address the challenges of new technology introduction and to include occupational health and safety in decisions about implementing new technology. However, there is no current guidance for livestock producers, and a production-oriented cost model may not account for injury and illness. Developing a comprehensive decision-making toolkit that accounts for occupational health will enable livestock stakeholders to implement technologies that enhance economic goals and the health of workers. Since there are questions limiting implementation in all barns, needleless injectors are a timely and industry-relevant test case for developing and applying this toolkit.

The need for ergonomic interventions has been identified for agriculture in general [17, 54] and within the pig industry in particular [5], but few have been systematically evaluated. Intervention research is the primary avenue for research-to-practice translation [16]. However, as an industry with narrow financial margins [5], pork production requires intervention evaluations that include economic comparisons in order to motivate adoption of safety interventions. Although changes in musculoskeletal exposure are a valuable measure of an intervention's effects, any efforts to effectively implement and promote OH&S interventions in the swine industry need to demonstrate sensitivity to the intensive production context; consider the work culture and attitudes of users; examine potential unintended consequences; and demonstrate a return on investment in order to motivate implementation.

Safety interventions (such as a needleless injector) are often multifaceted and introduced into complex environments; personal factors such as self-efficacy and skills, as well as organizational factors such as management support and device accessibility, can impact the degree to which an intervention is implemented [39]. Because of the intensive context of animal production, economic evaluations of new methods like needleless injectors have held a lot of research interest in the swine industry [55]. Integrating ergonomics, worker health, and safety with corporate goals like quality and productivity is key to motivating adoption of safer practices and equipment [11]. However, economic evaluations of ergonomic interventions are rare [56], highlighting the need for a more comprehensive approach to workplace intervention evaluation.

A comprehensive decision-making tool needs to incorporate all the dimensions of successful implementation, as well as those which influence decision-making regarding adopting a new technology. For example, Schell et al. describe nine main areas of influence for public health intervention sustainability: “Political Support, Funding Stability, Partnerships, Organizational Capacity, Program Evaluation, Program Adaptation, Communications, Public Health Impacts, and Strategic Planning” [57]. The framework demonstrates how relationships between various collaborations influence planning and evaluations, overall health impact, and longevity of such interventions and seems likely to also apply to occupational health since it is a component of public health. When aligned with engineering designs and goals of the organization from the outset, ergonomic interventions are reported to result in effective lessening of symptoms, lost work days, and claims for injuries during work [58], as well as better business performance [59, 60]. However, effective impact of ergonomics (and OH&S) interventions requires communication and agreement among decision-makers; a suitable means of knowledge translation is therefore needed to ensure a common stance among stakeholders [58, 61, 62]. In a production-driven private enterprise, cost-benefit analysis is a common metric and could form the foundation for discussion and adoption of new technology. In its final format, a toolkit to aid in this decision-making needs to be not only comprehensive but also understandable, usable, and accessible to knowledge users. For example, web-based decision-making tools are available for health care [63]. However, to our knowledge, this approach has not yet been applied to OH&S in intensive livestock production.

## 6. Limitations

While this project aims to fill a specific gap in knowledge, it also has some limitations. For example, the sampling strategy will likely include more large firms than small firms (proportional to the workforce population); however, this may provide fewer samples on the conditions of smaller firms. In the case that these smaller firms are very diverse, we may consider oversampling them to get a more robust estimate of costs in this group; this will help to identify the production levels which derive a net benefit from adopting new technology. While this study will account for loss of a total pig carcass from a broken needle, meat quality losses arising from abscess, lesions, or infection at the injection site will not be included.

## 7. Conclusion

The study described in this protocol will use comprehensive evaluation to determine whether needleless injectors have a net health/economic benefit and also promote a highly industry-relevant and applicable set of research methods (the decision-making toolkit) that will be useful in OH&S throughout the pork value chain and highly applicable to other industries. We anticipate the first users of this research will be the swine industry representatives involved in our stakeholder advisory group. This work will also be transferable to other animal care tasks in the agriculture industry, including dairy, cattle production, veterinary services, and equestrian work. In addition to these benefits to the industrial sector, this project will contribute to future research by developing a parsimonious suite of methods for comprehensive evaluation of occupational health and safety interventions.

## Abbreviations

MSD: Musculoskeletal disorder
sEMG: Surface electromyography
OH&S: Occupational health and safety
WCB: Workers Compensation Board
FTE: Full-time equivalents
ANOVA: Analysis of Variance
APDF: Amplitude probability distribution function
HAL: Hand Activity Level
KT: Knowledge translation.

## Data Access

As per the policies of the University of Saskatchewan, the data from this study will be stored for five years after the last publication.
